# Factors associated with maternal mortality in Malawi: application of the three delays model

**DOI:** 10.1186/s12884-017-1406-5

**Published:** 2017-07-12

**Authors:** Florence Mgawadere, Regine Unkels, Abigail Kazembe, Nynke van den Broek

**Affiliations:** 1 Centre for Maternal and Newborn Health, Liverpool School of Tropical Medicine, Pembroke Place, Liverpool, L3 5QA UK; 2 Kamuzu College of Nursing, University of Malawi, Zomba, Malawi

**Keywords:** Three delays model, Maternal mortality, Maternal death review, Contributing factors

## Abstract

**Background:**

The three delays model proposes that maternal mortality is associated with delays in: 1) deciding to seek care; 2) reaching the healthcare facility; and 3) receiving care. Previously, the majority of women who died were reported to have experienced type 1 and 2 delays. With increased coverage of healthcare services, we sought to explore the relative contribution of each type of delay.

**Method:**

151 maternal deaths were identified during a 12-month reproductive age mortality survey (RAMOS) conducted in Malawi; verbal autopsy and facility-based medical record reviews were conducted to obtain details about the circumstances surrounding each death. Using the three delays framework, data were analysed for women who had; 1) died at a healthcare facility, 2) died at home but had previously accessed care and 3) died at home and had not accessed care.

**Results:**

62.2% (94/151) of maternal deaths occurred in a healthcare facility and a further 21.2% (32/151) of mothers died at home after they had accessed care at a healthcare facility. More than half of all women who died at a healthcare facility (52.1%) had experienced more than one type of delay. Type 3 delays were the most significant delay for women who died at a healthcare facility or women who died at home after they had accessed care, and was identified in 96.8% of cases. Type 2 delays were experienced by 59.6% and type 1 delays by 39.7% of all women. Long waiting hours before receiving treatment at a healthcare facility, multiple delays at the time of admission, shortage of drugs, non-availability and incompetence of skilled staff were some of the major causes of type 3 delays. Distance to a healthcare facility was the main problem resulting in type 2 delays.

**Conclusion:**

The majority of women do try to reach health services when an emergency occurs, but type 3 delays present a major problem. Improving quality of care at healthcare facility level will help reduce maternal mortality.

## Background

An estimated 303,000 women die during pregnancy, childbirth and the puerperium each year. The vast majority (99%) of these deaths occur in low- and middle-income countries and specifically in sub-Saharan Africa and South Asia [1]. Ending preventable maternal deaths continues to be one of the most important goals internationally – the target for the Millennium Development Goal (MDG) 5a was to reduce the maternal mortality ratio (MMR) by three quarters between 1990 and 2015 [2]. Recently, a new target was set which aims for less than 70 maternal deaths per 100,000 live births by the year 2030 [2].

Evidence-based effective interventions to reduce maternal morbidity and mortality include; access to Skilled Birth Attendance (SBA) during childbirth and Emergency Obstetric Care (EmOC) when a woman experiences obstetric complications [3]. Efforts to reach the MDG target in Malawi failed despite a significant increase in institutional deliveries and skilled attendance at birth from 51 and 55% in 1992 to 91 and 90% in 2015, respectively [4], Most maternal deaths are still attributed to direct obstetric causes (haemorrhage, sepsis, complications of abortion and hypertensive disorders [5]. Although access to health care and coverage has improved, it is important that the available care is of good quality defined by six characteristics; safety, effectiveness, patient-centredness, timeliness, equitable and efficient [6].

The three delays model developed by Thaddeus and Maine is the most common framework used to evaluate the circumstances surrounding a maternal death [7]. These are 1) delay in deciding to seek care, 2) delay in reaching a healthcare facility 3) delay in receiving care at the healthcare facility. Type 1 delays are influenced by the factors involved in decision-making; sociocultural factors; financial and opportunity costs. Factors such as distance to the nearest healthcare facility, travel time, availability and cost of transportation; road conditions contribute to type 2 delays. Type 3 delays include factors affecting the speed with which effective care is provided once a woman reaches a healthcare facility; shortages of supplies, equipment, and trained personnel; competence of available personnel and quality of care.

Until recently, type 1 and 2 delays were documented to be the main problems [8–10]. Global statistics now show increased access to, and availability of, care during pregnancy and birth and therefore this pattern may have changed [11]. It is important to document where delays occur as interventions for each type of delay are different and need to be prioritised according to identified need to accelerate further reduction in the number of preventable maternal deaths. The three delays model helps to determine where improvements can be made to save the lives of women and babies. We used this framework to analyse the delays associated with all maternal deaths that occurred in one year in a large rural district in Malawi.

## Methods

### Setting

Malawi is one of the eighteen countries in sub-Saharan Africa with a very high MMR (634 per 100,000 live births) [1]. Malawi has total a population of 13.1 million with females comprising 51% of the total population [12] of which 45% are in the reproductive age bracket (15–49 years). Administratively, the country is divided into three regions: the northern, central and southern regions, which are further divided into 28 districts, six in the northern region, nine in the central region and 13 in the southern region. Health services are provided at three levels: primary, secondary and tertiary. At primary level, services are delivered through rural hospitals, health centres, health posts, outreach clinics and through community health initiatives.

Mangochi is one of the largest districts in southern Malawi covering an area of 6273 km^2^. The district has a population of 916,274 with an estimated 207,868 women of reproductive age (WRA) and 42,496 births per year [13].

The district has 46 healthcare facilities; four hospitals (secondary level), 39 health centres (primary level) and 3 dispensaries (primary level) [14]. Mangochi District Hospital acts as the main referral hospital for all lower level healthcare facilities in the district. Over half of all healthcare facilities (30, 65.2%) are operated by the Ministry of Health (MoH) providing free services. Sixteen (34.8%) healthcare facilities are private and charge a service fee.

The district has relatively poor reproductive health indices in comparison to the national averages. The proportion of women which have skilled attendance at birth is lower compared with the national average (68.5% and 71.4% respectively) [14].

### Identification of maternal deaths

In this study, we included all 151 maternal deaths (MD) that occurred in the district during one year. All deaths that occurred either at health facility or community level were identified through a prospective Reproductive Age Mortality Study (RAMOS) conducted in the district from Dec 2011 to Nov 2012 inclusive [15].

A RAMOS uses multiple sources of data (triangulation) to identify all deaths among WRA to ensure the most comprehensive results possible [15]. Sources may vary depending on country setting and may include vital registration, health facility records, burial records, and interviews with family members, coroners’ offices, the police, public morgues, traditional birth attendants and others. Most often, a RAMOS is conducted retrospectively, but may also be done prospectively if a population of WRA is monitored over time and all deaths are reviewed as they occur. A RAMOS is conducted in two phases. The first phase involves the identification of all deaths among WRA in a population using multiple sources. These sources of information are used to create a list of the deceased WRA. In the second phase, all deaths are investigated (using verbal autopsy, health facility reports or medical records, death certificates with medical cause and interview with household members and relatives) to ascertain if they were pregnant.

In this study, all deaths of WRA at participating healthcare facilities occurring in the maternity ward or any other ward where women aged 15–49 years accessed care were identified by healthcare providers and reported to the research team. In addition, hospital registers as well as the mortuary registers were reviewed. The purpose of the latter was to capture deaths which were directly transferred to the mortuary, either after a road traffic accident or deaths occurring at home and where relatives required storage of the body awaiting burial. At community level, deaths among WRA were identified via heads of households, village leaders, traditional healers, burial sites, village registers, traditional birth attendants and police stations. In addition, health surveillance assistants (HSA), community-based healthcare workers in Malawi, expected to aggregate data on deaths occurring in the community and share information on any recorded deaths.

To ensure data quality and to avoid missing any deaths, the research staff visited all 46 participating healthcare facilities once a month, cross-checked all registers and checked findings with the respective healthcare providers. At community level, quarterly review meetings were held with the HSAs and village leaders to identify any deaths not yet reported. At the end of every month, lists from the two data sources, (health facilities and community), were compared and any duplicates removed. All deaths among WRA were reviewed by the research team. During this study, 424 deaths among WRA were identified and of these 151 deaths were classified as maternal deaths, defined as:

*“The death of a woman while pregnant or within 42 days of termination of pregnancy irrespective of the duration and the site of the pregnancy, from any cause related to or aggravated by the pregnancy or its management but not from accidental or incidental causes.”* [16].

Experienced, trained research staff visited all households in which a maternal death had occurred to interview all persons (including Traditional Birth Attendants, neighbours and relatives) who had knowledge of the woman’s illness and death using a standard verbal autopsy questionnaire. In addition, all maternal deaths were reviewed by an expert panel to assess cause of and factors associated with each maternal death.

Most women died at a healthcare facility (62.3%, 94/151), four of these women died during referral from one healthcare facility to another. Just over a third of all maternal deaths (31.7%, 57/151) occurred outside a healthcare facility of which 51 died at home, four in transit to a healthcare facility and two at a traditional healer.

### Review of maternal deaths

Trained researchers participating in the RAMOS study conducted a community-based verbal autopsy for all 151 maternal deaths regardless of place of death using standardised procedures and forms. Families and friends were interviewed to obtain details about the medical and social circumstances surrounding each death using the WHO verbal autopsy tool [17] with additional questions on:

- Care received during pregnancy and delivery
- Decision making process regarding seeking health care (if care accessed)
- History of journey to healthcare facility (if the woman accessed a healthcare facility)
- Referral status and treatment at the referral facility
- Reasons for seeking care
- Reasons for not seeking care for those who died at home

Case histories were created for each maternal death using all documents available including any case notes, all verbal autopsy information, interviews with medical personnel and/or information obtained via facility-based maternal death review (available for 86 women).

### Application of the three delays model

Information was reviewed by two experienced researchers (a midwife and an obstetrician) independently. Using the thematic framework approach, key themes were identified and used to develop a standardised framework for analysis to identify any delays experienced (Table 1) [18]. One or more delays may have occurred in each case. All cases were then jointly reviewed. In case of disagreement, a third experienced researcher reviewed the case information separately and agreement was reached by consensus as to whether or not of any of the three types of delay were experienced in each case resulting in maternal death (four cases, 2.6%). Analysis was conducted separately for 1) women who sought care at a healthcare facility and died at the facility, 2) women who had sought care at a healthcare facility but later died at home, and, 3) women who did not seek care and died at home.

**Table 1:** Framework for analysis used to identify type of delay for maternal deaths

Delay 1 – Decision to seek care	Delay 2 – Reaching care	Delay 3 – Receiving care
Low status (woman not financially independent or husband not available)	No healthcare facility in the area. (takes more than one hour to reach healthcare facility)	Long waiting time before treatment was received (more than 30 min from the time of arrival to time of being assessed or receiving treatment)
Lack of awareness of obstetric complications	Long travel time from home to a healthcare facility (more than an hour)	Shortage of equipment and supplies
Nearest healthcare facility is more than 1 km away	Cost of transportation	Wrong assessment of risk, wrong diagnosis, wrong treatment
Uneventful previous home delivery	Poor road condition or terrain	Shortage of healthcare providers
The family has insufficient money	Visited a traditional healer or traditional birth attendant first	Lack of competence or skills among the available healthcare providers
Poor experience of previous health care received at a healthcare facility		Healthcare provider unavailable
Perceived poor quality of care at a the healthcare facility		Inadequate referral system, (ambulances not available, no fuel, breakdown and use of public transport)
Avoiding admission and long stay (more than two days) at a healthcare facility		Lack of treatment guidelines e.g. Pre-eclampsia, PPH, manual removal of placenta etc

### Ethical approval

Ethical approval for this study was obtained from the Liverpool School of Tropical Medicine in the United Kingdom and the College of Medicine Ethics Committee in Malawi.

## Results

### Socio demographic characteristics

In the 12-month period, a total of 424 women aged between 15 and 49 years died; 151 of which were identified as maternal deaths (35.6%). The mean (SD) age at the time of death was 27 (6.8) years and the age range was 15–45 years. Compared with women who died at home, women who died at a health facility were younger (mean (SD) age 26 (6.5) years versus 34 (6.9) years), more likely to have attended school (79% vs 19%) and more likely to be giving birth for the first time (primiparity 50% vs 3.5%). Almost half of the deceased women did not have any formal education. The proportion of adolescent maternal deaths (15–19 years) was 15.2% (23/151) and the number maternal deaths over 40 years of age was 9 (6.0%). Eighty-five percent of the women who died were married. The majority of women were multiparous (55.6%, parity 2 to 4; 11.9%, parity ≥5) and 32.4% were primiparas.

### Women who died at a healthcare facility

Out of the 94 women who sought care and died in a healthcare facility, the majority of women died in the postnatal (39/94, 41.5%) or labour ward (22/94, 23.4%). However, almost a quarter of women (21/94, 22.3%) died during admission procedures, 2 in the operating theatre, 6 in the female ward and 4 during referral.

More than half of all women (49/94, 52.1%) had experienced more than one type of delay. Almost all women who died experienced type 3 delays (91/94, 96.8%). The least frequent delay were type 1 delays but this was still experienced by 39.4% of women (37/94), 49 women (52.1%) experienced all three types of delays, and, type 2 delays were noted in 56 women (59.6%).

### Reasons for type 3 delays

Most women who died at a healthcare facility experienced a type 3 delay (91/94, 96.8%). Almost all women (89/94, 94.7%) had delayed treatment on admission (Table 2). Forty-five women (50.6%), were attended to within 30–60 min of arrival to the facility, 31 (34.8%) after 1–2 h and 13 women (14.6%) died before they were attended to.

**Table 2:** Type and reason for delays identified for maternal deaths (*n* = 151)

Type of delay	Reason for the delay	Number of women	Percentage (%)^a^
Women who died at a healthcare facility (*n* = 94)			
Type 3 Delay (*n* = 91)	Long waiting time before treatment at a healthcare facility	89	94.7
	Shortage of equipment and supplies	62	66
	Wrong assessment of risk, wrong diagnosis, wrong treatment	48	51.1
	Inadequate referral system	36	40.4
	Lack of competence on EmOC among the available personnel	27	28.7
	Staff unavailable	22	23.4
	Shortage of trained staff	15	16
	Lack of treatment guidelines	12	12.7
Type 2 Delay (*n* = 56)	Long travel time from home to a healthcare facility	49	52.1
	High cost of transportation	37	39.4
	Poor road condition or terrain, difficulty crossing rivers	17	18.1
	Visited a traditional healer first or traditional birth attendant	14	25.5
	Lack of a healthcare facility in the area	7	7.4
Type 1 Delay (*n* = 37)	Lack of awareness of obstetric complications	20	21.3
	Low income of the family	16	17
	Visited a traditional healer	14	14.9
	Do not want to stay long at a healthcare facility	13	13.8
	Low status of a woman (not financially empowered or husband not available)	11	11.7
	Long distance to a healthcare facility	7	7.4
	Bad experience with previous health care	5	5.3
	Uneventful home delivery previously	4	4.2
	Perceived poor quality of care at a healthcare facility	3	3.1
Women who had accessed care at a healthcare facility but died at home (*n* = 32)			
Type 3 Delay (*n* = 30)	Long waiting time before treatment at a healthcare facility	26	81.3
	Shortage of drugs	17	53.1
	Staff unavailable	13	40.6
	Shortage of trained staff	7	21.9
	Inadequate referral system	5	15.6
Type 2 Delay (*n* = 11)	Long travel time from home to a healthcare facility	7	21.8
	Lack of a healthcare facility in the area	5	15.6
	Cost of transportation	4	12.5
	Poor road condition or terrain, difficulty crossing rivers	3	9.3
Type 1 Delay (*n* = 9)	Lack of awareness of obstetric complications	7	21.9
	Low income of the family	6	18.8
	Bad experience with previous health care	6	18.8
	Perceived poor quality of care at a healthcare facility	5	15.6
	Low status of a woman (not financially empowered or husband not available)	4	12.5
	Uneventful previous home delivery	3	9.4
Women who did not access care and died at home (*n* = 25)			
Type 1 Delay	Perceived poor quality of care at a healthcare facility	16	64
	Lack of awareness of obstetric complications	13	52
	Uneventful previous home delivery	9	36
	Bad experience with the previous health care	8	32
	Low status of a woman (not financially empowered or husband not available)	8	32
	Long distance to a healthcare facility	4	16
	Low financial status of the family	3	12

^a^More than one type of delay possible for each maternal death, presented in order of frequency

Shortage of equipment, drugs and supplies was the second most frequent cause of type 3 delays, (62/91, 63.1%). This included lack of antibiotics (35/62, 56.5%), magnesium sulphate (22/62, 35.5%) and lack of blood in the blood bank (13/62, 21.0%). Three facilities had an insufficient supply of gloves and healthcare providers reserved the gloves for assessing women in the second stage of labour only. As a result, seven women experienced prolonged labour which was not detected and subsequently died of sepsis. Two women who suffered from ruptured uterus experienced delays in undergoing laparotomy due the lack of sterilised surgical instruments. All equipment had to be sterilised in a different healthcare facility 40 km away as the sterilizer was not functional in the first hospital. One woman died before going into theatre and the other died on theatre table before the operation commenced.

Forty-eight women (51.1%) were wrongly assessed and received the wrong treatment. Thirty-six, (38.3%) could not be referred. Of these, 13 women were delayed because the ambulance was not working so they used alternative public transport but died on arrival at the referral healthcare facility. For 11 women, an ambulance arrived two hours after it was requested and for 6 women there was evidence that healthcare providers had delayed calling an ambulance for referral. Four women died on the way to a referral hospital which was more than two hours’ drive away. Two women died at a health centre while waiting for transport to a referral hospital.

### Pattern of delay by healthcare facility level

Delays due to shortages of equipment, incorrect risk assessment, incorrect diagnosis and treatment were experienced equally by women both at hospital and health centre level. More women (75/89, 84.3%) experienced long waiting times before receiving treatment at a hospital compared to a health centre (24/89, 27.0%). However, more delays were experienced by women at a health centre (Table 3).

**Table 3:** Common type 3 delays experienced by women at hospitals and health (*n* = 94) centres

Reasons for Type 3 Delay	No of women at hospital level	Number of women at health centre level	Total^a^
Long waiting time before treatment at a healthcare facility	75	24	89^a^
Shortage of equipment and supplies	45	50	62^a^
Wrong assessment of risk, wrong diagnosis, wrong treatment	20	36	48^a^
Inadequate referral system	2	34	36
Lack of competence on EmOC among the available personnel	3	24	27
Staff unavailable	4	21	22^a^
Shortage of trained staff	9	12	15^a^
Lack of treatment guidelines	0	12	12

^a^Some women experienced the same delay at both the hospital and health centre level, therefore the total exceeds 94

### Reasons for type 2 delays

Delays in reaching a healthcare facility due to long distance was reported for 49 women (52.1%). An ox cart, the slowest mode of transport, was used in six cases. A total of 37 (39.4%) women were delayed due to high transport cost. It took an average of two hours to secure adequate money for transport when the decision to seek care was made.

### Reasons for type 1 delays

Just over a third of women (37/94, 39.3%) who accessed care experienced type 1 delays. Fourteen women (14.9%) consulted traditional healers before accessing care (7 suffered from dizziness, 4 premature labour, 2 vomiting in pregnancy and 1 retained placenta) before visiting a healthcare facility. Thirteen women (13.8%) delayed seeking professional care because they did not want to stay in the labour ward for a long period (Table 2). Nine of these women were multigravidas who waited for established labour at home and arrived at the healthcare facility late and in the second stage of labour. The remaining 4 were primigravidas who reported with either prolonged premature rupture of membranes or obstructed labour because they had been wrongly advised by older guardians/relatives who felt they were not in established labour and had kept the women at home as they were thought to have exaggerated the signs and symptoms of labour because of lack of previous birth experience.

### Women who died at home

A total of 57 women (57/151, 37.7%) died at home. We divided these women into two groups: a) women who had sought care and subsequently died at home (*n* = 32) and b) women who did not seek care and died at home (*n* = 25).

#### Delays experienced by women who sought care and later died at home

Over half (32/57, 56.1%) of the women who died at home, had previously sought care at either a health centre (22) or a hospital (10) before they died. The main reasons for seeking care were; obstetric danger signs as outlined in Table 4. Four women had attended a healthcare facility for delivery.

**Table 4:** Reasons for seeking care at a healthcare facility for women who had accessed care but died at home (*n* = 32)

Reason	Number of women (%)	Place of care	
		Hospital (%)	Health centre (%)
Foul smelling discharge after delivery	8 (25)	1 (10)	7 (31.8)
Dizziness during pregnancy	5 (15.6)	3 (30)	2 (9.2)
Bleeding in pregnancy	4 (12.5)	1 (10)	3 (13.6)
PPH	4 (12.5)	1 (10)	3 (13.6)
Delivery	4 (12.5)	2 (20)	2 (9.2)
Malaria in pregnancy	2 (6.3)	0 (0)	2 (9.2)
One week postnatal check up	2 (6.3)	1 (10)	1 (4.5)
Loss of body weight	1 (3.1)	1 (10)	0 (0.0)
ART supply	1 (3.1)	0 (0)	1 (4.5)
Antenatal care	1 (3.1)	0	1 (4.5)
Total	32	10 (100)	22 (100)

Long waiting hours before treatment at a healthcare facility and shortage of drugs were the leading causes of type 3 delays, (26 and 17 women respectively). Thirteen women waited for up to one hour, 10 for more than an hour and 3 spent the whole day at a healthcare facility before they were attended to. Fifteen (15) women were told to buy their own drugs and 2 women were sent away because the healthcare facility had no drugs.

Long distances and non-availability of a healthcare facility within their catchment area were the major reason for type 2 delays (Table 2). Lack of awareness of obstetric complications, low financial status and perceived poor quality of care reported by the deceased women relatives. Three women reported not going to a nearby healthcare facility (7 km) but going to a private facility (17 km) away because of poor quality of care at the nearest healthcare facility.

#### Delays experienced by women who did not seek care and died at home

Out of 57 maternal deaths that occurred outside a healthcare facility, only 25 (25/151, 16.6%) occurred at home and these women who had not accessed care in the period leading up to their deaths. These women did not seek care all. Perceived poor quality of care at the healthcare facility, lack of awareness of obstetric complications, poor financial status and uneventful previous delivery were the main reasons given by the women’s families for not seeking care (Table 2). Pallor, headache, vomiting, fever and dizziness, antepartum haemorrhage in early and late pregnancy which are all potential danger signs but also considered normal signs of pregnancy because they were common among pregnant women in the district.

## Discussion

Among 151 maternal deaths identified in Mangochi, a rural district in Malawi over a period of one year, only 16.6% of women who died had not accessed care at a healthcare facility in the period leading up to their deaths. Our findings show that the women who died experienced all three types of delay: 1) the decision to seek care, 2) reaching care and 3) receiving care. However, type 3 delays were the most frequently encountered and were identified in 96.8% of all maternal deaths. Any type of delay has been shown to be associated with poorer outcomes, [19] but type 3 delays are typically indicative of substandard quality of care at a healthcare facility [7, 20].

Contributing factors included long waiting times before receiving any type of assessment or treatment at a healthcare facility, non-availability of essential drugs, consumables or equipment, lack of skilled personnel, missed and incorrect diagnoses, delayed or incorrect treatment. Health system failures have been identified as a major contributing factor to maternal deaths and the delay in or inability to ensure timely referral was also noted in this study [21–24]. Facility-based maternal death reviews in Malawi revealed that 20 out of 28 maternal deaths were associated with healthcare provider factors which were avoidable and administrative failure [25]. Similarly, in a study from Ethiopia, 88% of maternal deaths (*n* = 34) reviewed at healthcare facility level were associated with medical failures [26]. In a district-based audit of maternal deaths in Indonesia, 60% of maternal deaths (*n* = 130) were identified as having experienced type 3 delays [27]. In South Africa, a confidential enquiry into maternal deaths revealed that poor clinical assessment, delays in referral, failure to follow standard protocols and not responding to abnormalities in the monitoring of patients were the most common avoidable healthcare provider factors [28]. Poor quality of care at healthcare facility level experiences of poor care during previous visits were reported as some of the reasons for type 1 delay by women.

Many healthcare facilities in low- and middle-income settings are still chronically under-resourced and unable to offer safe, effective care to women with obstetric complications. In this study, the lack of essential equipment, sufficient number of competent healthcare providers, and stock-out of essential drugs resulted in women who accessed care not receiving timely, effective care. It is important that healthcare providers are able to work in a well-functioning health system where good quality of care can be provided. Adegoke et al. described a well-functioning system as one that provides an “enabling environment” which includes sufficient human and financial resources, essential drugs, and the necessary equipment [29]. Skills-based training to ensure all healthcare providers are competent in skilled birth attendance, emergency obstetric and neonatal care is also important [30, 31].

In this study, we found there were delays in the necessary referrals of mothers from one healthcare facility to another due to the lack of emergency transport. During the study period, there were severe fuel shortages in Malawi [32]. In Gambia, ambulances were used for alternative purposes and relatives were asked to hire transport [33]. Other studies have similarly reported an association between maternal deaths and lack of effective referral mechanisms between healthcare facilities [32, 34]. Other recent studies from low- and middle-income countries have similarly documented that non-availability of quality emergency obstetric care remains a major problem [28, 35, 36].

This study shows that comparatively few women experienced type 1 delays. Barriers related to healthcare-seeking behaviour such as economic status, distance to a healthcare facility, women’s autonomy and knowledge of danger signs continue to lead to delays in the decision and departure to seek care. However, negative past experiences and expectations of poor quality of care are highlighted as important factors contributing to type 1 delay. Poor quality care influences women’s decision-making and mitigates against timely access to care [10]. This study was conducted in Malawi and may not reflect the pattern of delays across other countries with persistently high maternal maternity ratios. However, the findings are important and can be used to improve the quality of care in similar settings. The three delays framework continues to be important and shuld be used to identify where delays occur in other settings.

## Conclusion

The majority of women who died during pregnancy, childbirth or the puerperium had accessed care at a healthcare facility during the time preceding their deaths. Effective emergency care is dependent upon a healthcare provider’s ability to recognise that an abnormal condition exists, that the condition has a level of severity warranting intervention and that an intervention is available to treat the condition. Care must be provided in a timely and woman-friendly manner if lives are to be saved. Improving availability and quality of emergency obstetric care services is needed if maternal deaths are to be prevented.

## Abbreviations

EmOC: Emergency Obstetric Care
MD: Maternal Deaths
MDG: Millennium Development Goal
MMR: Maternal Mortality Ratio
RAMOS: Reproductive Age Mortality Study
SBA: Skilled Birth Attendance
WRA: Women of Reproductive Age

## References

[1] World Health Organization, UNICEF, UNFPA, World Bank, United Nations Population Division (2015). Trends in maternal mortality 1990 to 2015: estimates by the WHO, UNICEF, UNFPA, the World Bank and the United Nations population division.

[2] World Health Organization (2015). Health 2015: from MDGs-millennium development goals to SDGs-sustainable development goals.

[3] World Health Organization and Aga Khan University. Essential Interventions, Commodities and Guidelines for Reproductive, Maternal, Newborn and Child Health: A global review of the key interventions related to Reproductive, Maternal, Newborn and Child Health (RMNCH). Geneva: PMNCH, World Health Organization; 2011.

[4] Malawi National Statistical Office, ICF Macro. Malawi Demographic and Health Survey, 2010. Zomba, Malawi; Calverton, MD: National Statistics Office; ICF Macro; 2011..

[5] Mgawadere F, Unkels R, van den Broek N (2016). Assigning cause of maternal death: a comparison of findings by a facility-based review team, an expert panel using the new ICD-MM cause classification and a computer-based program (INTERVA-4). BJOG.

[6] Institute of Medicine (2001). Crossing the quality chasm.

[7] Thaddeus S, Maine D (1994). Too far to walk: maternal mortality in context. Soc Sci Med.

[8] Somé DT, Sombié I, Meda N (2013). How decision for seeking maternal care is made–a qualitative study in two rural medical districts of Burkina Faso. Reprod Health.

[9] Filippi V, Richard F, Lange I, Ouattara F (2009). Identifying barriers from home to the appropriate hospital through near-miss audits in developing countries. Best Prac Res Cl Ob.

[10] Killewo J, Anwar I, Bashir I, Yunus M, Chakraborty J (2006). Perceived delay in healthcare-seeking for episodes of serious illness and its implications for safe motherhood interventions in rural Bangladesh. J Health Popul Nutr.

[11] World Health Organization (2015). World health statistics 2015.

[12] Malawi National Statistical Office (2008). 2008 population and housing census.

[13] Malawi National Statistics Office. Population and Housing Census. Malawi National Statistics Office: Zomba; 2008.

[14] Malawi Ministry of Health (2012). Health management information system (HMIS) 2011–2012.

[15] Mgawadere F, Adegoke A (2017). Van den Broek. Reproductive age mortality survey (RAMOS) in Mangochi, Malawi. BMC Pregn Childb.

[16] World Health Organization (1992). International statistical classification of diseases and related health problems, tenth revision.

[17] World Health Organization (2007). Verbal autopsy standards: ascertaining and attributing causes of deaths.

[18] Ritchie J, Lewis J (2003). Qualitative research practice: a guide for social science students and researchers.

[19] Ebrahimian A, Seyedin H, Jamshidi-Orak R, Masoumi G (2014). Exploring factors affecting emergency medical services staffs’ decision about transporting medical patients to medical facilities. Emerg Med Int.

[20] van den Broek NR, Graham WJ (2009). Quality of care for maternal and newborn health: the neglected agenda. BJOG.

[21] Combs Thorsen V, Sundby J, Malata A (2012). Piecing together the maternal death puzzle through narratives: the three delays model revisited. PLoS One.

[22] Orji EO, Ojofeitimi EO, Esimai AO, Adejuyigbe E, Adeyemi AB, Owolabi OO (2006). Assessment of delays in receiving delivery care at a tertiary healthcare delivery centre in Nigeria. J Obstet Gynaec..

[23] Stekelenburg J, Roosmalen JV (2002). The maternal mortality review meeting: experiences from Kalabo District hospital, Zambia. Trop Doc.

[24] Walraven G, Telfer M, Rowley J, Ronsmans C (2000). Maternal mortality in rural Gambia: levels, causes and contributing factors. Bull World Health Organ.

[25] Kongnyuy EJ, Mlava G, van den Broek N (2009). Facility-based maternal death review in three districts in the central region of Malawi: an analysis of causes and characteristics of maternal deaths. Women Health Iss.

[26] Hailu S, Enqueselassi F, Berhan Y (2009). Health facility-based maternal death audit in Tigray, Ethiopia. Ethiopian J Health Dev.

[27] Supratikto G, Wirth ME, Archadi E, Cohen S, Ronsmans C (2002). A district-based audit of the causes and circumstances of maternal deaths in South Kalimantan, Indonesia. Bull World Health Organ.

[28] Moodley J (2010). Maternal deaths associated with eclampsia in South Africa: lessons to learn from the confidential enquiries into maternal deaths, 2005-2007. SAMJ S Afr Med J.

[29] Adegoke AA, Hofman JJ, Kongnyuy E, van den Broek N (2011). Monitoring and evaluation of skilled birth attendance: a proposed new framework. Midwifery.

[30] Grady K, Ameh C, Adegoke A, Kongnyuy E, Dornan J, Falconer T (2011). Improving essential obstetric and newborn care in resource-poor countries. J Obstet Gynaec.

[31] Ameh CA, Kerr R, Madaj B, Mdegela M, Kana T, Jones S (2016). Knowledge and skills of healthcare providers in sub-Saharan Africa and Asia before and after competency-based training in emergency obstetric and early newborn care. PLoS One.

[32] Barnes-Josiah D, Myntti C, Augustin A (1998). The “three delays” as a framework for examining maternal mortality in Haiti. Soc Sci Med.

[33] Cham M, Sundby J, Vangen S (2005). Maternal mortality in the rural Gambia, a qualitative study on access to emergency obstetric care. Reprod Health.

[34] Fatusi A, Ijadunola K (2003). National Study on essential obstetric care facilities in Nigeria: technical report.

[35] Essendi H, Mills S, Fotso JC (2011). Barriers to formal emergency obstetric care services’ utilization. J Urban Health.

[36] Ameh CA, Msuya S, Hofman J, Raven J, Mathai M, van den Broek N (2012). Status of emergency obstetric Care in six Developing Countries Five Years before the MDG targets for maternal and newborn health. PLoS One.

